# Acute Respiratory Distress Syndrome Caused by Occupational Exposure to Waterproofing Spray: A Case Report and Literature Review

**DOI:** 10.3389/fpubh.2022.830429

**Published:** 2022-02-25

**Authors:** Meng Fu, Chun-Mei Feng, Le-Jie Cao, Xiao-Wen Hu, Qi-xia Xu, Huai-Ling Xia, Zi-Mei Ji, Na-Na Hu, Wang Xie, Yuan Fang, Da-Qing Xia, Jun-Qiang Zhang

**Affiliations:** ^1^Department of Respiratory and Critical Care Medicine, The First Affiliated Hospital of University of Science and Technology of China (Anhui Provincial Hospital), Hefei, China; ^2^Anhui Province Key Laboratory of Medical Physics and Technology, Institute of Health and Medical Technology, Hefei Institutes of Physical Science, Chinese Academy of Sciences, Hefei, China; ^3^Department of Respiratory and Critical Care Medicine, Second Affiliated Hospital of Anhui Medical University, Hefei, China

**Keywords:** ARDS, occupational exposure, waterproofing spray, mNGS, glucocorticoids, case report

## Abstract

**Background:**

Acute respiratory distress syndrome (ARDS) is a serious respiratory disease, caused by severe infection, trauma, shock, inhalation of harmful gases and poisons and presented with acute-onset and high mortality. Timely and accurate identification will be helpful to the treatment and prognosis of ARDS cases. Herein, we report a case of ARDS caused by occupational exposure to waterproofing spray. To our knowledge, inhalation of waterproofing spray is an uncommon cause of ARDS, and what makes our case special is that we ruled out concurrent infections with some pathogens by using metagenomic next-generation sequencing (mNGS) as an auxiliary diagnosis, which presents the most comprehensive etiological examination of similar reports.

**Case Presentation:**

A previously healthy 25 years old delivery man developed hyperpyrexia, chest tightness, cough and expectoration. The symptoms occurred and gradually exacerbated after exposure to a waterproofing spray. The chest computed tomography (CT) finding showed diffuse ground glass and infiltrative shadows in both lungs. The diagnosis of ARDS related to waterproofing spray was established on the basis of comprehensive differential diagnosis and etiological examination. The patient achieved good curative effect after proper systemic glucocorticoid therapy.

**Conclusions:**

The diagnosis and differential diagnosis of acute respiratory failure for outdoor workers, such as delivery drivers or hikers, should be considered whether toxic aerosol exposure exists from daily contacts. The case can educate the public that more attention should be paid to avoid exposure to these chemicals by aerosols/ingestion mode and some preventive strategies should be taken in occupational environment. The treatment effect of glucocorticoids is significant in ARDS patients with general chemical damage caused by inhaling toxic gases and substances.

## Introduction

Acute respiratory distress syndrome (ARDS) is characterized by refractory hypoxemia and progressive respiratory failure, caused by severe infection, trauma, shock, inhalation of harmful gases and poisons (1–3). Timely identification and diagnosis, accurate assessment of disease severity, and early initiation of therapy will improve prognosis (4). Integrated therapeutic strategies of ARDS include treatment of primary disease, prone position ventilation, lung protective ventilation strategy, extracorporeal membrane oxygenation, short-term of neuromuscular blockers and glucocorticoid (3, 5–7). Prognosis of patients suffering with ARDS depends on the primary diseases, complications, effect of treatment and drug-related adverse reactions.

Inhalation injury is an acute respiratory tract damage, caused by direct thermal injury, carbon monoxide poisoning, or toxic chemical inhalants including mist, fumes, and gases (8). ARDS caused by inhalation injury of occupational exposure should paid more attention to, especially for outdoor workers, such as delivery man or cleaner. Most of the time they need to work outside, even in rainy days. Therefore, it is necessary to make waterproofing process on footwear during outdoor activities. Waterproofing products can be impervious to water or dirt by using coat textile fabric, leather or solid surfaces. These products consist of three key parts: a water repellent, a solvent and a propellant (9). The inescapable truth is that these products may lead to acute lung injury after frequent and continuous contact (10–12). Herein, we report a case of ARDS caused by occupational exposure to waterproofing spray. This is a rare case which excluded the possibility complicated with specific pathogens by metagenomic next-generation sequencing (mNGS) as an auxiliary diagnosis, which presents the most comprehensive etiological examination of similar reports.

## Case Presentation

On November 7, 2020, a previously-healthy 25 years old delivery man was sent to the emergency room with hyperpyrexia and chest tightness for about 22 h, accompanied by cough and expectoration. Through case history inquiry, we known that he applied a homebred waterproofing spray (about 100 mL) to his shoes in a bathroom 6 h prior to symptom onset, being exposed to terrible smells and poor ventilation room. He smelt pungent odor when inhaled directly some of the spray. Additionally, he smoked 10 cigarettes a day for 5 years. He had no history of drinking and neurological or psychiatric disorders. Family members of the patient were in good health. There was no history of hereditary diseases, preexisting sensitivity or pulmonary disease, such as asthma, pneumonia, tuberculosis, cardiovascular diseases, infectious diseases, and surgical interventions. The patient with low flow oxygen presented stable vital signs by a bedside ECG monitor [heart rate (HR) 71/min, blood pressure 126/69 mmHg, SpO_2_ 97% (91% in room air), respiratory rate (RR) 22/min]. General physical examination and specific check-up for the rest body system revealed no abnormality. Listening to the chest with a stethoscope revealed diminished respiration of bilateral lung.

**Figure 1:**
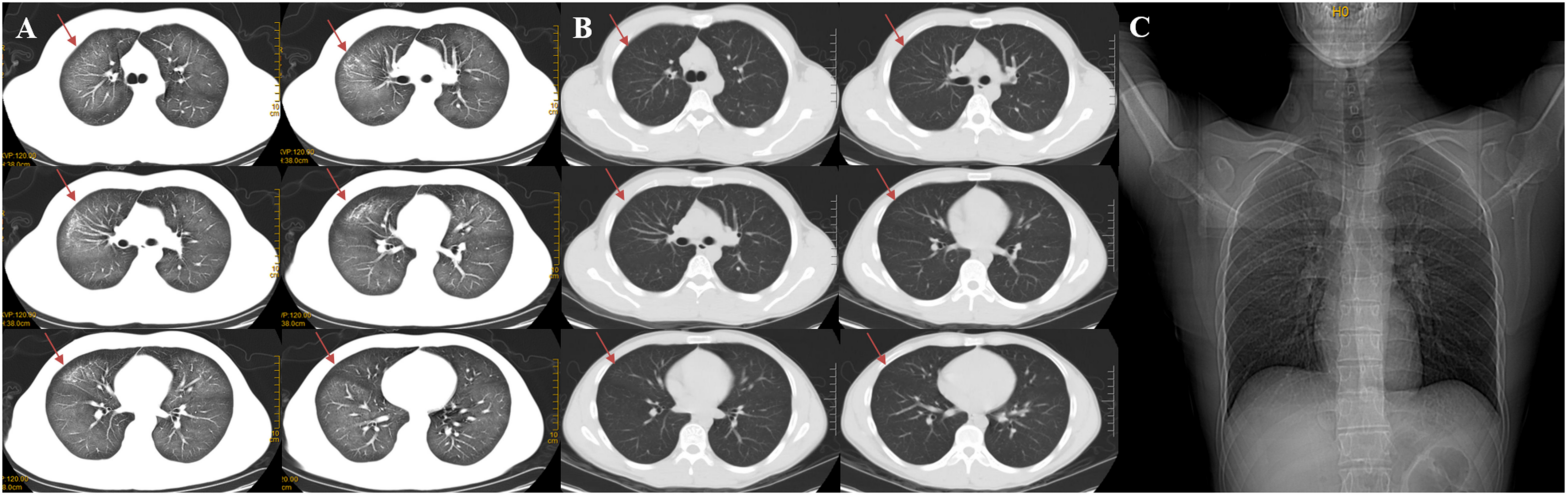
Changes of chest CT before **(A)** and after treatment **(B)**, a CT contrast of marked absorption of the lesion after 3 days of treatment. Admission chest X-ray of the patient **(C)**. **(A)** The chest CT scan demonstrated diffuse ground-glass opacity and infiltrative shadows. **(B)** After 3 days of using prednisone acetate, the chest HRCT revealed marked absorption of the pulmonary lesions. Red arrows show that the lesions in both lungs were absorbed. **(C)** Admission Chest X-ray of the patient demonstrated a decreased transparency in both lung field, and there was no signs of cardiomegaly and pleural effusion.

On admission, the routine blood test revealed total leucocyte count of 32.35 × 10^9^/L, of which neutrophile granulocyte count of 29.25 × 10^9^/L (90.4%), and procalcitonin (PCT) level of 8.66 ng/ml. C-reaction protein (CRP) level of 12.3 mg/L. There is no obvious abnormality of his blood serum chemistries and fibrin d-dimmer test, shown in Table 1. Blood gas analysis revealed PH 7.4, pO_2_ 66 mmHg, pCO_2_ 34 mmHg, HCO3^−^ 20 mmol/L, BE −1 mmol/L. PaO_2_/FIO_2_ = 220 (Table 1). His lung CT scan images revealed diffuse ground glass and infiltrative shadows (Figure 1A). But there were no evidences of immunosuppression and pathogens with sputum culture and blood serum test, such as bacteria, fungus, EB virus, CMV virus and so on. His antinuclear and vasculitis antibodies tests were normal. Electrocardiogram showed sinus rhythm. Admission chest x-ray of the patient demonstrated a decreased transparency in both lung field, and there were no signs of cardiomegaly and pleural effusion (Figure 1C). We also used fluorescence bronchoscope to obtain bronchoalveolar alveolar lavage fluid (BALF) for mNGS. The results of the mNGS were compared with four microbial genome reference sequence databases downloaded from the National Center for Biotechnology Information, which included the whole genome sequence of 1,798 DNA viruses, 6,350 bacteria, and 1,604 fungi and 234 parasites genome sequences associated with human infection. The results of mNGS exhibited *Dialister pneumosintes* (sequence number 46) and *Dialister invisus* (sequence number 36) which can often isolate from the mouths of animals and even humans (13, 14). They are usually considered as conditional pathogens of immunocompromised individuals and rarely seen in immunocompetent patients, so these pathogens were considered to be contaminating or colonizing bacteria (Table 2).

**Table 1:** Laboratory data of the patient after admission.

**Laboratory test**	**Results**	**Reference value**
**Complete bloodcount**
WBC	32.35	3.50–9.50
Neut	29.25	1.80–6.30
Lym	1.39	1.10–3.20
Mon	1.68	0.10–0.60
Eos	0.00	0.02–0.52
Bas	0.03	0.00–0.06
RBC	4.77	4.30–5.80
Hb	148	130–175
Hct	0.442	0.400–0.500
PLT	253	125–350
**Serologic tests**
CRP	12.3	0.0–10.0
PCT	8.66	0.00–0.10
d-dimer	0.28	0.00–0.50
**Blood gas analysis**
PH	7.40	7.35–7.45
pO_2_	66	80–100
pCO_2_	34	35–45
HCO3^−^	20	22–28
BE	−1	−3 to +3
PaO_2_/FIO_2_	220	400–500

*WBC (×10^9^/L), whitebloodcells; Neut, neutrophils; Lym, lymphocytes; Mon, monocytes; Eos, eosinophils; Bas, basophils; RBC (×10^9^/L), redblood cells; Hb (g/L), hemoglobin; Hct, hematocrit; PLT (×10^9^/L), platelets; CRP (mg/L), C-reactive protein; PCT (μg/L), Procalcitonin; G test, Fungi 1-3β glucan test; pCO_2_ (mmHg), partial pressure of carbondioxide; pO_2_ (mmHg), partial pressure of oxygen; HCO3^−^ (mmol/L), bicarbonate; BE (mmol/L), baseexcess*.

**Table 2:** Etiological examination results.

**Etiological examination**	**Results**	**Reference value**
COVID-19 DNA	–	–
CMV DNA	–	–
**EBV antibody**
Ig A &Ig M	–	–
Ig G	+	–
Mpn IgM	–	–
Cpn IgM	–	–
Lpn IgM	–	–
Rickettsia IgM	–	–
INFA&INFB IgM	–	–
RSV IgM	–	–
PIV IgM	–	–
ADV IgM	–	–
Sputum culture	–	–
Sputum smear for AFB	–	–
BALF mNGS	*Dialister pneumosintes* (46^*^)	
	*Dialister invisus* (36^*^)	

*–, negative; +, positive; COVID-2019, Corona Virus Disease-2019; CMV, Cytomegalovirus; EBV, Epstein-Barr virus; Mpn, Mycoplasma pneumonia; Cpn, Chlamydia pneumonia; Lpn, Legionella pneumophila; INFA, Influenza A Virus; INFB, Influenza A Virus; RSV, Respiratory syncytial virus; PIV, Parainfluenza virus; ADV, Adenovirus; AFB, acid-fast bacillus; BALF, Bronchoalveolar lavage fluid; mNGS, Metagenomic next-generation sequencing*.

* *The number of mNGS squences*.

**Figure 2:**
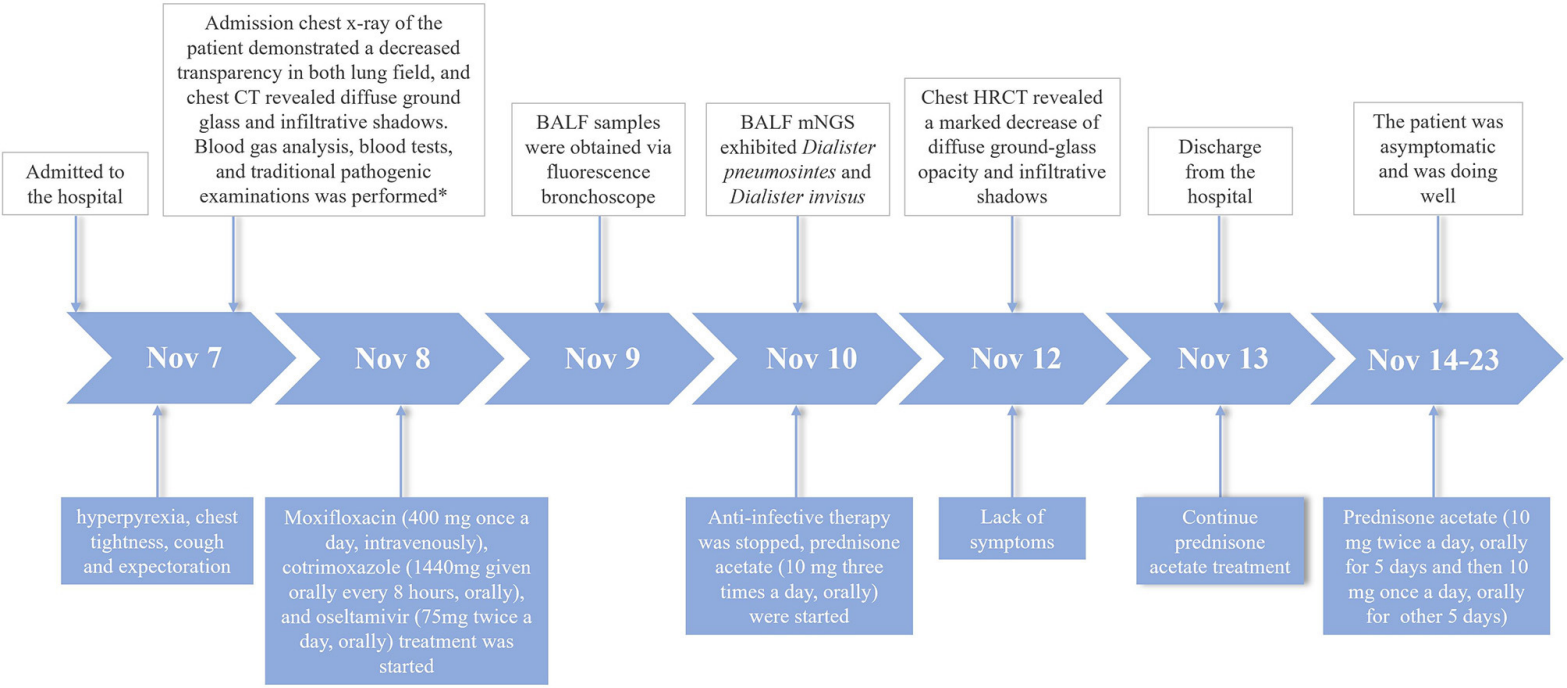
Case history of the patient with ARDS caused by occupational exposure to waterproofing spray. *The blood tests included complete bloodcount, serologic tests (c-reactive protein, procalcitonin, d-dimer); Traditional pathogenic examinations included sputum culture, nucleic acids of respiratory pathogens, sputum smear for acid-fast bacillus, and laboratory investigations of respiratory pathogens serum antibody.

Immediately on admission, based on the present and past history, age, clinical manifestations, physical examination and comprehensive auxiliary examinations, such as arterial blood gas analysis, a cardiogenic pulmonary edema was excluded and a preliminary diagnosis of mild ARDS was established. The patient was treated with moxifloxacin (400 mg given intravenously once a day), Cotrimoxazole (1,440 mg given orally every 8 h), oseltamivir (75 mg given orally twice a day), and terbutaline 1 mg (oxygen atomizing inhalation twice a day). We stopped anti-infective therapy after mNGS test, on account of lacking of etiological evidence. The recommendations from evidence-based medicine point out that a low dose of methylprednisolone 0.5–1 mg/kg body weight/d for mild ARDS (5). The total dosages of methylprednisolone were calculated based on the bodyweight of the patient (54 kg). And then the dosage of methylprednisolone (0.5 mg/kg body weight/d × 54 kg = 27 mg/d) was converted into the equivalent dosage of prednisone (27/4 × 5 = 33.75 mg/d). Subsequently, prednisone acetate (10 mg given orally three times a day) were started. The patient was afebrile and felt better after corticosteroid therapy. After 3 days of using prednisone acetate, the chest HRCT revealed a marked decrease of diffuse ground-glass opacity and infiltrative shadows (Figure 1B). The changes of neutrophil count, neutrophil proportion and white blood cell count had a continuously declining trend, which finally were close to normal. We decided to discharge the patient, and continued prednisone acetate for 10 days (10 mg given orally twice a day for 5 days and then 10 mg given orally once a day for 5 days). The patient treatment process is shown in Figure 2. During 12 weeks of follow-up, the patient was asymptomatic and was doing well. CT scans showed normal parenchyma of the lungs. Further follow-up observation is underway to research long term prognosis of ARDS patients involved in toxic inhalation.

## Discussion

We reported the case of a 25-year-old man diagnosed as ARDS just due to inhale toxic aerosol and ruled out concurrent infections with some pathogens by using metagenomic next-generation sequencing (mNGS) as an auxiliary diagnosis. ARDS and acute lung injury (ALI) were confirmed according to Berlin criteria and the American-European Consensus Conference on ARDS, respectively (15). In this case, we given the diagnosis of mild ARDS according to ARDS Berlin's criteria. The diagnosis was based on following diagnostic criteria: ① Symptoms of the patient appeared in ~6 h after using of the waterproofing spray, and exacerbated in the second day (<1 week). ② The CT scan revealed diffuse ground-glass opacity and infiltrative shadows, and there were no evidence of cardiogenic factors causing the symptoms and radiographic results. ③ The oxygenation index was from 200 to 300 (PaO_2_/FIO_2_ = 220). The inhalation of waterproofing spray was confirmed as the causative factor according to the evidence of comprehensive etiological examination while the evidence of microbial pathogenic background proved to be non-existent by mNGS. Rapid development of mNGS in recent years showed promising and satisfying application in medical microbiology. mNGS is a high-throughput sequencing technology that has broken the limit of traditional pathogen detection methods and allows for hypothesis-free, culture-independent pathogen detection directly from biological samples, including cerebral spinal fluid, blood, urine, and BALF samples (16). ALI/ARDS can result from various pathologies including sepsis, microbial infection, trauma or ischemia/reperfusion, with rapid progress and high mortality. Bacterial and viral respiratory infections (including secondary bacterial infections after an initial viral infection) are the most common etiological factors of respiratory failure including ALI and ARDS. It is crucial to figure out whether it is initiated by pathogenic bacteria based on the results of mNGS during the diagnosis and treatment processes of ARDS, which is linked to antibiotic administration, changes in therapy, or discontinuation of antibiotic therapy. In this case, the differential diagnoses just from the chest imaging manifestation should include viral pneumonia, hypersensitivity, drug-induced damage, acute eosinophilic types of pneumonia, and opportunistic infections (17). Thus, we performed mNGS to identify possible pathogens of the patient, and the results suggested that the lung lesions were uncorrelated with infectious factors.

The pathophysiologic hallmark of ARDS is alteration of increasing pulmonary vascular leakage, leading to pulmonary edema, in which protein-rich fluids flood the alveolar spaces, impair gas exchange, and culminate in respiratory failure (18). The pathogeny of ARDS in this case may be consistent with the typical pattern of chemical pneumonitis, in which infiltration of neutrophils into the alveoli and pulmonary interstitium leads to the acute inflammatory response that generally occurs 4–6 h after the insulting event. Chemical pneumonitis, as a common complication, often occurs after inhalation of toxic fumes or gases. The initial pathological events confined to the distal airway are results of cellular toxicity of the inhaled agent which disturb the impermeability of alveolar capillary interface. Severe pulmonary edema will inevitably occur and gas exchange will also be impaired due to absence of intact alveolar interface. The pulmonary edema presents a rapidly progressive development after a latent period and the severity tends to depend on inhaled dose. From mild alveolar infiltrates to diffuse alveolar damage eventually leads to ARDS (19). According to previous study, even a single exposure can lead to long term sequelae like reactive airway dysfunction syndrome (RADS), bronchiolitis obliterans, or bronchiolitis obliterans with organizing pneumonia (BOOP) (20, 21).

The first similar case was reported in the US, and a consumer who contacted with 1,1,1-trichloroethane-based products suffered from acute respiratory failure (20, 22). Approximately 20 reports about various waterproofing agents resulting in different health effects have been described in the past 38 years (23). The complex composition of spray products and the toxicity of waterproofing aerosol spray are associated with fluorinated compounds (24, 25). A smaller particle size will allow the product to reach deep into the lungs, even to the alveoli and respiratory bronchioles that are covered by a thin liquid film of lung surfactant. The toxicity will be reduced if the particle size of fluoropolymer fumes increased (26). Aerosol particles with a diameter 10 μm aerosol have been confirmed to be risk factors for chemical pneumonitis (27). The waterproof sprays involved in this case are mainly composed of fluorocarbon resin, synergist, organic solvent, diluent, heptane solvent, which are consistent with previous researches. This finding can remind us that we should pay more attention to products which contain fluorinated compounds in daily life.

A systemic corticosteroid is usually administrated for the treatment of ARDS (28). Moreover, timely inhaled corticosteroids and beta-2 agonists may slow the progression by reducing lung inflammation and enhancing alveolar fluid clearance in ARDS patients (29). Only single-center study and small randomized trial demonstrated a certain effect of different dose of glucocorticoids in ARDS (5, 30). In our case, low-dose prednisone acetate played an important role in the treatment of waterproofing spray-related ARDS. However, more multicenter trials of high-dose glucocorticoids used in ARDS patients are restricted, because the increasing doses of glucocorticoids are associated with adverse reactions (28). Effective pharmacologic treatments need to be further explored directly targeting lung injury in ARDS patients (29).

## Conclusion

We should consider whether toxic gases and substances are inhaled for outdoor workers who present with unexplained respiratory symptoms during the daily diagnosis and treatment process. Glucocorticoids have been shown to be effective to the inhaled chemical damage. For the public, especially for outdoor workers, precaution should be taken to avoid damage of occupational exposure. Therefore, the popularity of targeted occupational health care education still needs to be strengthened.

## Data Availability Statement

The original contributions presented in the study are included in the article/Supplementary Material, further inquiries can be directed to the corresponding author/s.

## Ethics Statement

The studies involving human participants were reviewed and approved by the Medical Ethics Committee of Anhui Provincial Hospital. The patients/participants provided their written informed consent to participate in this study. Written informed consent was obtained from the individual(s) for the publication of any potentially identifiable images or data included in this article.

## Author Contributions

MF, C-MF, L-JC, and J-QZ contributed to the conception and presentation of the case report. L-JC, J-QZ, and D-QX provided clinical expertise and interpretations. L-JC and J-QZ performed the fluorescence bronchoscope to collect BALF sample. MF and J-QZ analyzed the sequencing of mNGS. L-JC, J-QZ, D-QX, MF, and Z-MJ overall management and treat the patient. MF wrote the first draft of the report. C-MF contributed to manuscript revision. All authors contributed to the article and approved the submitted version.

## Funding

This work was supported by the Anhui Provincial Key Clinical Specialty Discipline Construction Program (2021szdzk05).

## Conflict of Interest

The authors declare that the research was conducted in the absence of any commercial or financial relationships that could be construed as a potential conflict of interest.

## Publisher's Note

All claims expressed in this article are solely those of the authors and do not necessarily represent those of their affiliated organizations, or those of the publisher, the editors and the reviewers. Any product that may be evaluated in this article, or claim that may be made by its manufacturer, is not guaranteed or endorsed by the publisher.
