# Supplementary material for: Acute Respiratory Distress Syndrome Caused by Occupational Exposure to Waterproofing Spray: A Case Report and Literature Review
Source: Front Public Health. 2022 Feb 25;10:830429. doi: 10.3389/fpubh.2022.830429 (PMC8916539; doi:10.3389/fpubh.2022.830429)
Supplement: Supplementary file 1 [file Table_1.DOCX]

**Informed Consent Form**

**Patient’s consent for the publication of material relating to**

**them in the Frontiers in Public Health journals**

**To be completed by the corresponding author:**

Subject of article or photograph:

Name of author submitting material: Meng Fu

Corresponding author’s address: Lujiang Road no 17, Hefei, 230001, Anhui, China

Manuscript reference number:

**To be completed by the patient:**

I give my consent for all or any part of this material to appear in Frontiers in Public Health journals and all editions of Frontiers in Public Health journals, and any other works or products, in any form or medium.

I understand that:

- My name will not be published with the material and Frontiers in Public Health journals will endeavour to ensure my anonymity. However, despite Frontiers in Public Health journals’ best efforts, I understand that it is possible that somebody, for example members of my family or the health care staff who have looked after me, may recognise me from the image and/or the accompanying text.
- The uses of my material may include (without limitation) publication of the material in the print and electronic editions of Frontiers in Public Health journals, on websites, in sublicensed or reprinted editions (including foreign language editions), and in other works or products.
- I cannot revoke this consent once I have signed this consent form.
- This consent form will be retained by the corresponding author, and will not be sent to Frontiers in Public Health journals

Signed:

Date:

Print name:

If you are not the patient, what is your relationship to them?

Witness:

Date:
