# Supplementary figures and images for: Acute Respiratory Distress Syndrome Caused by Occupational Exposure to Waterproofing Spray: A Case Report and Literature Review
Source: Front Public Health. 2022 Feb 25;10:830429. doi: 10.3389/fpubh.2022.830429 (PMC8916539; doi:10.3389/fpubh.2022.830429)

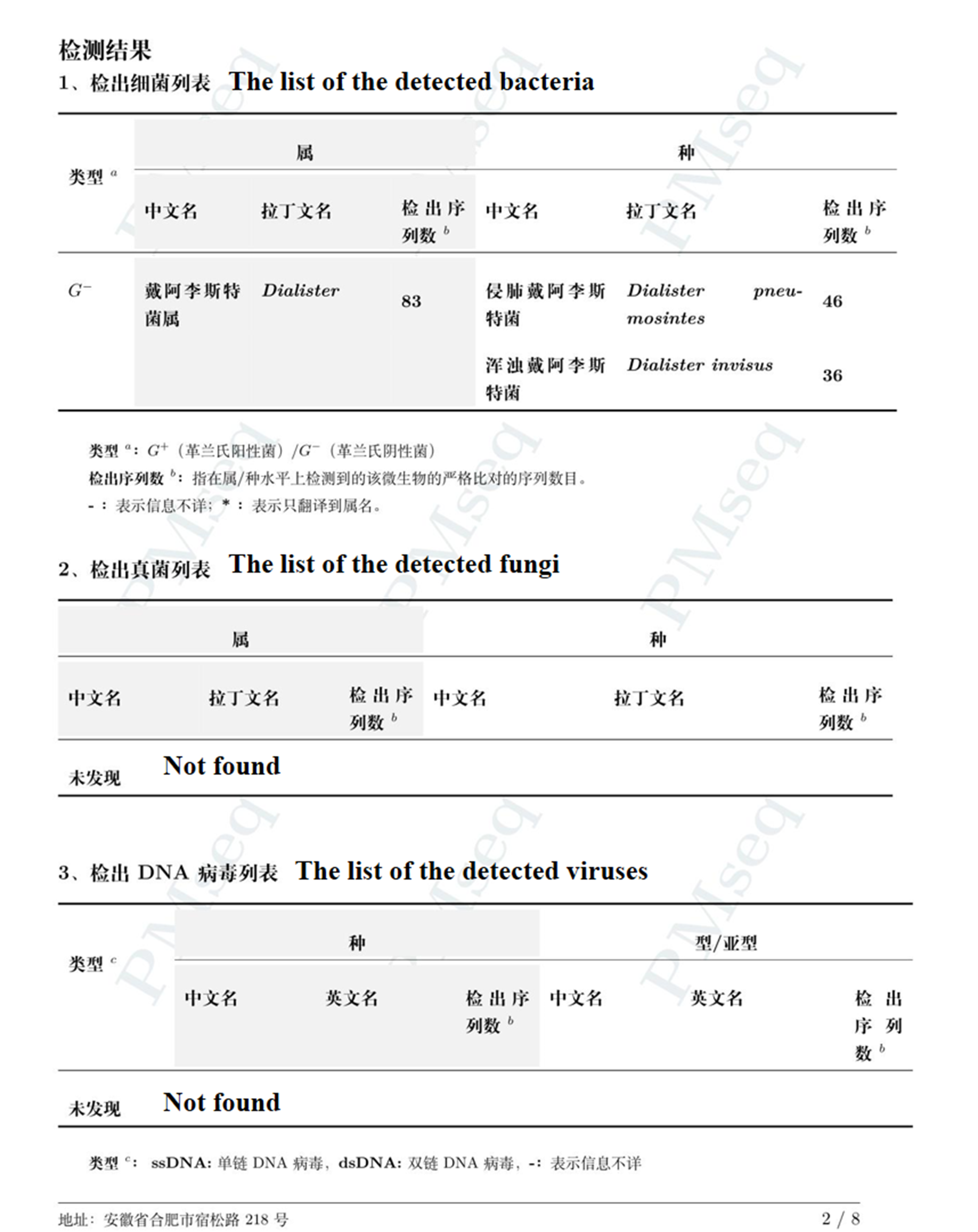

Supplement: Supplementary file 2 [file Image_1.PNG]

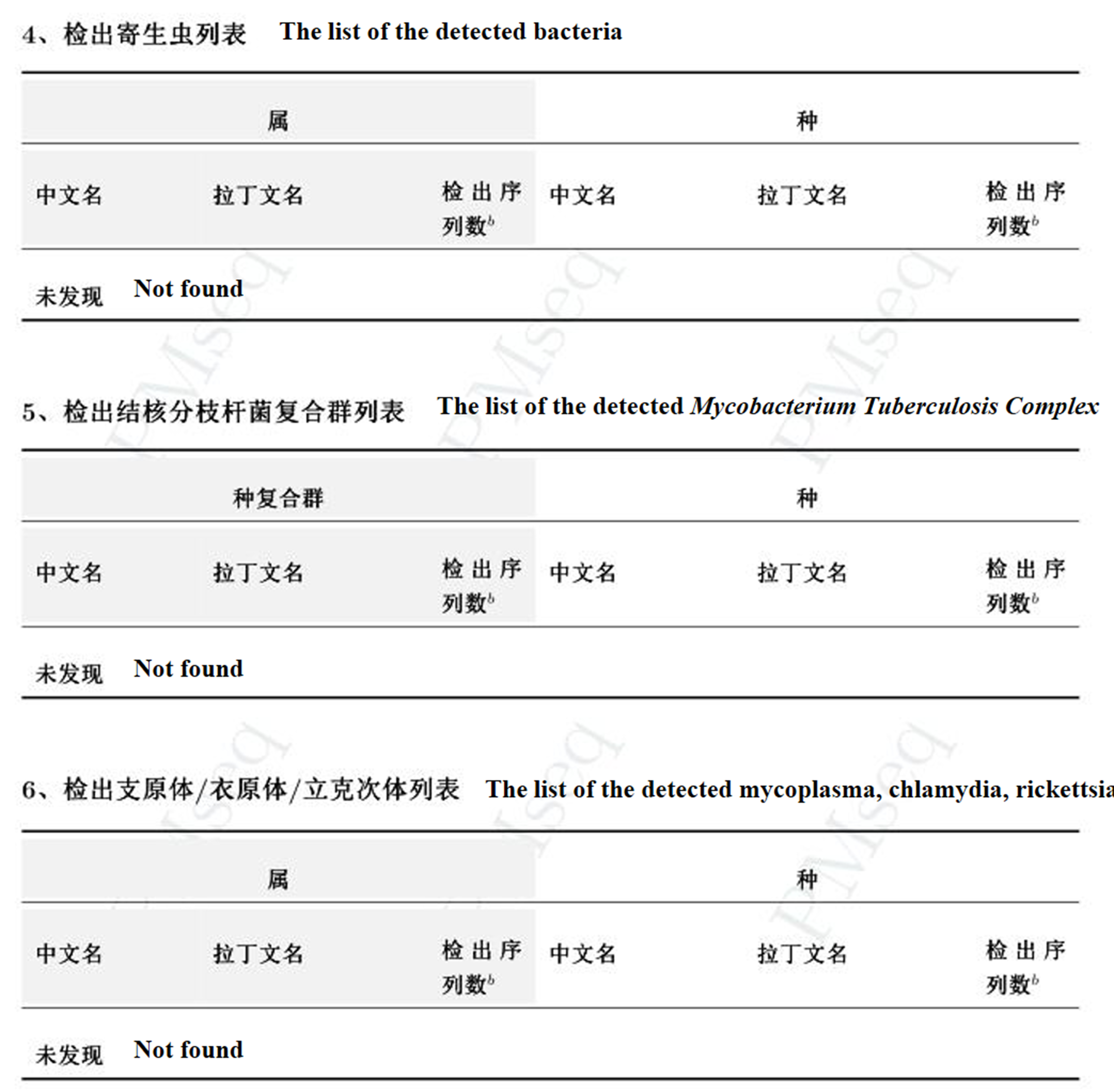

Supplement: Supplementary file 3 [file Image_2.PNG]
